# “Come and share your story and make everyone cry”: complicating service user educator storytelling in mental health professional education

**DOI:** 10.1007/s10459-022-10157-z

**Published:** 2022-09-08

**Authors:** Stephanie LeBlanc-Omstead, Elizabeth Anne Kinsella

**Affiliations:** 1 Health Professional Education, Faculty of Health Sciences, Western University, 1201 Western Rd., Elborn College, London, ON N6G 1H1 Canada; 2 Institute of Health Sciences Education, Faculty of Medicine and Health Sciences, McGill University, 1110 Pine Avenue West, Montreal, QC H3A 1A3 Canada; 3 School of Physical and Occupational Therapy, Faculty of Medicine and Health Sciences, McGill University, Montreal, QC H3A 1A3 Canada

**Keywords:** Epistemic injustice, Health professional education, Mad studies, Mad pedagogy, Mental health, Postcritical ethnography, Reflexivity, Service user involvement, Storytelling

## Abstract

It has become relatively common practice within health professional education to invite people who have used mental health and social care services (or *service user educators*) to share their stories with health professional learners and students. This paper reports on findings from a postcritical ethnographic study of the practice of service user involvement (SUI), in which we reflexively inquired into conceptualizations of service user educators’ knowledge contributions to health professional education in the accounts of both service user- and health professional educators. This research was conducted in response to recent calls for greater scrutiny surrounding the risks, challenges, and complexities inherent in involving service users in health professional education spaces. ‘Story/telling’ was identified as a pronounced overarching construct in our analysis, which focuses on participants’ reports of both the obvious and more subtle tensions and complexities they experience in relation to storytelling as a predominant tool or approach to SUI. Our findings are presented as three distinct, yet overlapping, themes related to these complexities or tensions: (a) performative expectations; (b) the invisible work of storytelling; and (c) broadening conceptualizations of service user educators’ knowledge. Our findings and discussion contribute to a growing body of literature which problematizes the uncritical solicitation of service user educators’ stories in health professional education and highlights the need for greater consideration of the emotional and epistemic labour expected of those who are invited to share their stories. This paper concludes with generative recommendations and reflexive prompts for health professional educators seeking to engage service user educators in health professional education through the practice of storytelling.

## Introduction

Health professional education programs have seen a rise in mental health service user involvement (SUI) initiatives over the past several decades (Beresford, 2002, 2003, 2005; Beresford & Croft, 1993; Happell et al., 2002; McKeown & Jones, 2014; McKeown, Malihi-Shoja & Downe, 2011; Repper & Breeze, 2007). As part of this commitment to SUI, it has now become relatively common practice within health professional education to invite people who have experience with mental health and social care services (hereafter, ‘service user educators’) to share their stories with health professional learners and students (de Bie, 2021). Indeed, storytelling—typically in the form of ad hoc guest lectures—remains the most common manner in which service user educators are involved in health professional education despite emerging growth in potential service user roles (e.g., curriculum co-production or design, content delivery, student selection/admission processes, and program evaluation) which have been elaborated in efforts to resist tokenistic involvement (de Bie, 2021; Happell & Bennetts, 2016; Sapouna, 2020; Soklaridis et al., 2020).

Storytelling from lived experience has long been central to the activist work and scholarship of Mad-identified people, psychiatric survivors and mental health service users (de Bie, 2021; Church, 1995; Costa et al., 2012; Crossley, 1999; Morrison, 2005; O’Donnell, Sapouna & Brosnan, 2019). In this context, storytelling has been posited as having potential to inspire radical change, identify and disrupt unequal relations of power, and redress injustices (Costa et al., 2012). Storytelling has also been used by service users to assert the power and value of experiential knowledge within and outside mental health systems (Voronka, 2015). When used as critical pedagogy, storytelling has the potential to reveal otherwise suppressed knowledge and make visible experiences of the world that are not typically represented by dominant knowledge paradigms (Razack, 1993). When used critically and intentionally service user educators’ stories stand to represent important ways of knowing mental distress beyond those typically represented within popular medicalized discourses of “descent into mental illness and heroic recovery” (de Bie, 2021, p. 1).

The solicitation of service user educators’ stories by the health professions represents an important shift—at least nominally—toward inclusion, wider acceptance of a diversity of knowers, and greater embrace of lived experience as a valid source of knowledge. Attention has been drawn to just how much of the literature related to mental health service users’ storytelling practices in health professional education contexts tends toward portrayals of its positive and uncomplicated aspects (de Bie, 2021; Happell & Bennetts, 2016). Findings from a recent critical interpretive review of this literature suggest that service user educators’ stories are most commonly used for the purposes of enhanced student engagement in active learning, cultivating empathy, complementing or critiquing professional knowledge, and offering real-life connections to course content and abstract theory (de Bie, 2021). Indeed, storytelling has been praised for its capacity to bring added value to the educational experience, provide unique insights into individual’s experiences of emotional distress (Felton & Stickley, 2004, p. 89), and promote transformative learning (Gidman, 2013; Troop & O’Riordan, 2017).

Although the potential for storytelling to be used as a tool for professional education and social change is remarkable, many service user educators, Mad scholars, activists, and critical health professionals have suggested that the use of storytelling in SUI remains insufficiently critiqued; pointing to the comparatively little attention that has been paid to its risks, challenges, and complexities (Brosnan, 2019; de Bie, 2021; Happell & Bennetts, 2016; Sapouna, 2020; Voronka & Grant, 2021). Razack (1993) cautions that storytelling should never be used uncritically as, “there are land mines strewn across the path wherever story-telling is used” (p. 56), and Sapouna (2020) warns that uncritical storytelling risks reinforcing and reproducing dominant biopsychosocial epistemologies of mental illness and neglects to disrupt the status quo. As such, the extent to which storytelling can be viewed as a productive means for conveying service user-produced knowledges in these spaces remains a contentious issue (Sapouna, 2020).

## Objectives

This paper responds to recent calls for greater scrutiny and more thoughtful consideration of the inclusions and exclusions, risks, challenges and complexities that arise when service user educators are engaged in health professional education contexts (Brosnan, 2019; de Bie, 2021; Ellaway, 2021; Happell & Bennetts, 2016; Sapouna, 2020; Voronka & Grant, 2021). In particular, we turn our focus to the ethical and epistemological tensions surrounding the pedagogical use of storytelling in SUI. Data analysis focused on participants’ reports of both the obvious and more subtle tensions and complexities they experienced in relation to storytelling as a predominant tool or approach to SUI. The findings of this study complicate taken for granted assumptions about the use of storytelling by service user educators in mental health professional education.

## Methodology

### Theoretical Framework

Our guiding theoretical frame draws on perspectives and conversations from the field of Mad Studies, Mad and critical pedagogies (Castrodale, 2017; Lather, 2001), and theories of epistemic injustice (Dotson, 2011; Fricker, 2007; Medina, 2012; Pohlhaus, 2012, 2014). Together, these critical theoretical lenses inform an understanding of service user involvement (SUI) as a pedagogical approach with potential to support service user educators’ epistemic contributions to the knowledge base of health and social care professions through involvement in the politicized and contested practice of health professional education.

Mad studies is a growing interdisciplinary field of social sciences and humanities research, which positions the ways of knowing, being and doing of Mad-identified, consumer/survivor/ex- patient (c/s/x), or service users as central and important in all matters related to understanding and responding to mental health (Beresford, 2005; Burstow, 2015; Burstow et al., 2014; Castrodale, 2017; Church, 1995; de Bie, 2021; LeFrancois, Menzies & Reaume, 2013; Reville, 2013). Drawing on Mad and critical pedagogical perspectives, we understand health professional education spaces to be complex discursive environments that shape and reproduce dominant social structures (Castrodale, 2017; Hooks, 2014; Lather, 1995). We view SUI as a critical pedagogical strategy to maximize inclusion of a plurality of perspectives through participatory learning (Castrodale, 2015).

However, informed by theories of epistemic injustice, we also acknowledge that within professional education classrooms, “all voices […] are not and cannot carry equal legitimacy, safety, and power” given present social structures (Lather, 1995, p. 172). Indeed, in the context of health professional education—with its foundations deeply rooted in biopsychosocial ways of knowing, relating, and responding to madness, mental distress and diversity—many service user educators experience epistemic marginalization related to both their knowledge, and their status as knowers. Service user educators’ knowledge—often shared through story or consultation—has yet to be regarded as equal to, or as legitimate as, professional, expert knowledge, and its exchange occurs between knowers with unequal epistemic power. Thus, epistemic injustice offers an important conceptual lens for examining SUI in health professional education in light of the imbalance of epistemic power and knowledge hierarchies at play.

### Postcritical ethnography

This paper reports on findings from a postcritical ethnographic study into SUI in mental health professional education within four occupational therapy programs across Ontario, Canada (LeBlanc-Omstead, 2021). Postcritical ethnography combines tenets drawn from poststructuralism with the critical ethnographic genre, constituting a research methodology, which aims to produce justice-centered discourses through the amplification of subjugated knowledges and stories (Anders, 2019; Noblit, 2004). While postcritical ethnographic research is not intended to be prescriptive, this methodology allows for projects to be framed in ways that might inform change (e.g., in practice or discourse), by way of inviting readers to, “consider what could be otherwise in inequitable relations but is not yet” (Anders, 2019, p. 1).

Like critical ethnographies, postcritical ethnographies take us “beneath surface appearances, disrupt the status quo, and unsettle both neutrality and taken-for-granted assumptions by bringing to light underlying and obscure operations of power and control” (Madison, 2011, p. 14). Where postcritical ethnographers make their methodological departure is in contextualizing their positionality within the research, in an attempt to make it more “accessible, transparent, and vulnerable to judgment and evaluation” (Madison, 2011, p. 19). Indeed, central to postcritical ethnography—as an approach to research as an ethical and political practice—is the importance of clarifying our own positionality as researchers within this work, and to avoid presenting our interpretations as though they have no ‘self’ (Anders, 2019; Olomo, 2006).

The first author approaches this work as a cisgender woman of white settler descent, as well as a Mad scholar and occupational therapist who has at various times (and often simultaneously) occupied the standpoints of service user- educator and health professional- educator. As a postcritical ethnographer concerned with the practice of SUI, she has been held, “both insider and outsider status”—simultaneously an onlooker, director, and member of the cast and her experiences in these roles provided the impetus for this research (Conquergood, 1992 as cited in Hart et el., 2017, p. 1766). The second author is a cisgender woman of white settler descent, and an ally of mental health service user educators. She is an academic of eighteen years whose scholarship has focused on epistemologies of practice, critical reflexivity, and interrogating taken-for-granted knowledge generating practices. She is interested in embodiment and epistemic injustice in health professions education and practice. In keeping with postcritical ethnographic methodology, we engaged an ongoing practice of critical reflexivity through: dialogue inspired by the first author’s reflexive journaling; critical interrogation of emerging insights; and dialogic debriefs with our other team members. Critical reflexivity was also employed as a means to navigate the first author’s ‘insider/outsider’ positionality.

The broad aims of the study were to (a) deepen understandings of the complex and varied experiences of SUI from the perspectives of both service user and health professional educators, and to (b) critically examine SUI as a pedagogical strategy for supporting the contribution of service user educators’ knowledge in health professional education. The research questions asked: (a) How do various stakeholders describe service user educators’ contributions to the education and knowledge base of future health professionals? And (b) To what extent does SUI as a practice support service user educators in contributing knowledge within the context of health professional education? The perspectives of both service user and health professional educators were collected in this study.

### Participants

Fourteen stakeholders engaged in the practice of mental health SUI participated in this study. All participants were engaged in SUI in one of the following capacities: (a) mental health service user educator involved in occupational therapy (n = 7) or other health and social care (n = 2) professional education program(s) in Ontario, Canada (total n = 9); or (b) health professional educator facilitating SUI in an occupational therapy professional education program (n = 5). Participants were recruited through the distribution of recruitment materials within four occupational therapy programs in Ontario, Canada, accompanied by a request for the circulation of materials to known service user educators with current and/or prior involvement in these programs. In addition, service user educators involved in other health and social care professional education programs were recruited through the announcement of the study in the Ontario Peer Development Initiative (OPDI) newsletter (considered to be highly visible to service user educators); and by word of mouth. Potential participants contacted the first author to express interest. Pseudonyms have been used in place of participants’ names, and any other potentially identifying information has been altered or removed in the presentation of the findings.

### Ethics

Approval to conduct this research was obtained from the Western University Non-Medical Research Ethics Board (NMREB).

### Data collection

Data sources for the study included in-depth recorded interviews, participant observation, and the first author’s autoethnographic and reflexive writing related to her own involvement in the practice of mental health SUI in the roles of service user educator and health professional educator. Each of the participants participated in an in-depth semi-structured interview that inquired into their experiences of SUI (e.g., their experiences as educators; reasons for becoming involved and/or soliciting involvement; perceived benefits and challenges), and other pertinent details related to their role(s) and context of involvement (e.g., recruitment/hiring practices; role title; remuneration; communicated involvement expectations or learning objectives). In addition to the first author’s firsthand involvement in the practice of SUI, she engaged in participant observation during two service user educators’ guest lectures in two separate occupational therapy programs.

### Data Analysis

Interviews with service user and health professional educators were transcribed verbatim. Data analysis of the interviews was led by the first author using a reflexive approach guided by Srivistava and Hopwood’s (2009) framework for analytic reflexivity. The following three questions were used to refine the research focus and integrate data: (1) What are the data telling us? (2) What is it we want to know? And, (3) what is the dialectical relationship between what the data are telling us and what we want to know? The first of these questions was also used to question our role as interpreters, by way of asking, “what [are] the data telling [us] that they might not tell someone else”? For instance, as previously noted, the first author identifies with the communities of both service user- and health professional- educators. It is possible that her firsthand experience in these roles, her affiliation to activist communities, and her critical analytic lens, resulted in an analysis that was acutely attuned to issues such as epistemic injustice and invisible, emotional labour.

According to Srivistava and Hopwood (2009) this reflexive analytic framework “might offer one of the many ways of writing yourself into the narrative without being self-indulgent or distracting from the purpose of research”; an endeavor consistent with a postcritical ethnographic research methodology. Quirkos, a qualitative data management software, was used to organize the data into visual and thematic representations. Other collected data (i.e., participant observation field notes and reflexive journal entries) were compiled and referred to regularly to help contextualize and inform interpretations arising throughout the analysis of the interviews. The second author contributed to the data analysis through a) discussion about the coded excerpts of the transcripts, b) dialogue meetings to discuss reflexive insights emerging during the data analysis process and c) regular meetings to explore evolving thematic representations of the data.

### Findings

‘Story/telling’ was identified as a pronounced overarching construct from our reflexive inquiry into participants’ conceptualizations of service user educators’ knowledge contributions to health professional education. Through our inquiry we came to appreciate that it is often the very qualities or aspects of service user educator storytelling garnering the most praise and attention, that upon closer examination can be revealed as sites of epistemic and/or ethical tension. Thus, informed by the critical theoretical frame described above, we identified three themes centered around participants’ accounts of the complexities and tensions they experienced related to SUI through storytelling: (a) performative expectations; (b) the invisible work of storytelling; and (c) broadening conceptualizations of service user educators’ knowledge. Participants’ accounts presented hereafter reflect both the widely accepted benefits and/or admirable features of service user storytelling (e.g., their unique capacity to evoke emotion), as well as the often overlooked complexities or risks associated with these (e.g., invisible, emotional labour).

### Performative expectations: “come and share your story and make everyone cry”

#### The power of stories

Service user educators’ stories were frequently described by participants as “powerful” (Sara, health professional educator) in their capacity to enhance learning, deepen student engagement, evoke emotion, leave a lasting impact on, or “strike a chord” (Lindsay, health professional educator) in their listeners. Rita (health professional educator) noted how learning from stories “stays with the students” long after they have forgotten the content of conventional lectures, explaining:

The messages are very powerful... students become very engaged and most have a point of reference… It's touching a chord because of their own position, and relationship to mental health issues. So, it's just one step less removed, and it really infiltrates, not only their thinking, but their being.

Lindsay (health professional educator) also shared this perspective, stating:

The students remember [service user educators’ stories] for the rest of their career. I can tell you every person with lived experience that came in to present to my program when I was doing my [health professional] Master’s. They were very powerful; but I can't remember every lecture.

The use of story as a powerful tool for effecting change and inspiring action toward “improving the system”—something Sara (health professional educator) described as an “advocacy objective”—was one of the most commonly cited motivations amongst participants for sharing, or soliciting, service user educators’ stories. Sara explained: “It becomes a very powerful tool when people, with this capacity to effectively share their story… express their story in a way that effects change,” and recalled the involvement of one service user educator whose, “career has become about using his story as a platform to help policy and service providers and frontline workers and decision makers think in different ways about trauma, and understanding trauma.”

Other participants spoke about the power of storytelling to challenge dominant narratives, or contribute to a diversity of stories. Heather (service user educator) explained that she tells her story because,

I think that the only way that the whole story can be—the whole mental health system and the outcomes—can be understood, is when you hear all of [the story]; not just part of it. And the only way you're going to hear all of it, is for myself and others to come forward and say, this happened and this is what the outcome was.

#### Performance metaphors and embodied performance

Our analysis suggested that the power of stories described by so many participants was related to more than their content, but perhaps also to the manner in which these stories were shared—or rather—performed. In contrast with conventional guest lectures, delivered by health professionals for instance, service user educators’ knowledge sharing through storytelling was often discussed by participants in relation to notions of performance—metaphorically, in an embodied sense, or as an unspoken requirement of SUI. Perceived performative expectations were revealed in service user educator participants’ frequent use of metaphors centered around conventional forms of performance or entertainment (e.g., a play, concert, or movie) to describe the knowledge they share through storytelling. For Kimberly (service user educator), stories help theoretical knowledge, course concepts or “textbook examples” to “come alive.” She drew parallels between hearing service user educators’ stories and seeing a play, rather than reading the screenplay:

I remember in high school when we had to read Shakespeare, and I'm like ‘oh, this is so boring’... but I can go to a Shakespearian play and love it. It's easier to hear something and see something, than it is to just pick up the words on a page.

Edward (service user educator) likened hearing a service user educator’s story to seeing a live concert: “What can I say? I'd rather see The Rolling Stones than read about them. As good as the writers are, and the interviews are great; when you see them, you get a rush.” Sally (service user educator) compared the dialogue with students following storytelling to speaking with an actor about their role in a movie, noting that it gave room for more inquiry and depth of understanding:

I think [with a] textbook... you hear a story; versus when I’m presenting the story about my life, [students] can ask deeper into it. If I’m going to watch [a movie], I'm going to watch it 10 times and I'm not going to get more information. But, if I speak to the woman who played it; played the role, or lived that life, I can understand the depth.

Others described perceived performative expectations in a more embodied sense, using the language of “modeling wellness” (Elliot, service user educator) or presenting themselves as “success stories” (George, service user educator). Elliot (service user educator) discussed that an important part of his role was demonstrating to students, “How well [service user educators] can be, how much insight we can have, and therefore, break down some of that stigma people have.” He further explained:

On an acute ward [health professionals] never see anybody when they're well. They only see them when they're unwell. So, in some ways I'm presenting wellness... They lose a bit of hope when they only see somebody at their worst... So, I always felt like I was modeling wellness. And, therefore, giving them hope. Giving them context that recovery is possible and probable.

Fred (service user educator) stated, “I wanted to share my story to give [health professional students] hope that people can change, and there are success stories out there…” He recalled:

I was definitely asked to share my personal story of going from homeless and suffering mental health and addiction issues to how I became housed, a business owner, working and traveling... you know, my story of changing my life around.

Closely linked to the embodied performance of wellness, was the notion that storytelling can be effectively used for the purpose of ‘humanizing’ those who experience mental distress. For Fred (service user educator), this meant helping students to, “see a wounded person, not a bad person.” And as Sally (service user educator) explained:

We can tell a bit of our story; they can see us as *real* people who are, you know, standing in the room with them... just seeing people for being who they are, and how they are, and not scary monsters.

Toward the goal of humanizing, other participants spoke about being asked to offer firsthand insights into *why* service user educators might engage in certain behaviours (especially those typically deemed ‘difficult’ by health professionals), in order to instill in students greater feelings of empathy, understanding and patience. Fred (service user educator) offered students “personal knowledge” of, “say, why [he] used to miss doctor's appointments” explaining that, “if [students] can understand how the client—what their life is like, what they've gone through, or how their day has been—then it would help [students] to better serve the client.” Glen (service user educator) described seeing “real value” in being able to “explain *why* [he] was doing things” during periods of significant mental distress and/or addiction, as well as presenting students with a contrasting image of himself “as [he is] now.”

#### Troubling performative expectations

While many participants acknowledged that story can be used as a powerful tool for enhancing student engagement, shifting attitudes, elucidating mental health-related concepts, and ‘humanizing’ experiences of mental distress, several problematized the expectation that SUI entail performative or evocative storytelling. Joel (service user educator) suggested that perceived expectations around storytelling can be internalized by service user educators as a pressure to possess and retell particularly compelling, engaging or entertaining stories:

We tend to judge ourselves... [For example] ‘Do we have what it takes to do X or Y or Z?’ and in this case, because of what this work requires, what it ends up being is, ‘well, have we been crazy enough? is our story outrageous enough?

Heather (service user educator) rejected the expectation that service user educators should be entertaining, saying, “I'm not there to entertain! …It's serious. It's people's lives!” And Glen (service user educator) underlined the potential for objectification in soliciting deeply personal service user educator stories for the purpose of education, stating: “Right, so what is it you're accessing when you're putting the service user ‘on stage’? I call it emotional pornography.” Some health professional educators described conflicted feelings about seeking service user educators’ stories for their evocative potential, or for the sake of enhanced student engagement. Sara (health professional educator) expressed having “mixed feelings” about soliciting personal stories:

Sometimes what [service user educators] are sharing is actually quite personal... They're sharing their experience of a loss sometimes... And, we're asking them to stand up in front of 60 strangers and share that. And I think that's really hard. Then we say to them, ‘Thank you very much, here's a gift card.’ And, something about that, at times, can feel quite... I don't know the right word for it, maybe... quite perverse... For lack of a better word, it feels a little perverse, to just say: “come and share your story and make everyone cry.”

### The invisible work of storytelling: “sharing your story can be very draining.”

#### Deciding which story/ies to tell?

Participants described a considerable amount of work involved in storytelling, which often began with composing a story and navigating the difficulties inherent in attempting to condense years, or decades, of lived experience into a single lecture or self-contained story. In Heather’s (service user educator) words: “…you're only given so much time, and how do you put 72 years into a 2-min talk? Or, 10 to 15-min talk? It’s hard.” Several participants described finding it difficult to reach decisions about which experiences should be incorporated into their stories, because these stories naturally evolved over time with the accumulation of new experiences, personal growth, and opportunities for self-reflection. In Sally’s (service user educator) words:

I'm not the same person today that I was 15 years ago; and the story that I told 15 years ago would be very different than the story I told today. Partly because of the interaction with the people that I tell that part of my life to.

To complicate matters, several participants described feelings of uncertainty about what service user educators ought to be sharing with health professional students in the first place. This may have been due in part to a lack of clearly communicated expectations between health professional academics and service user educators. Sara (health professional educator) offered this reflection: “Where I think things get awkward, is when… it's just, ‘come and share your story,’ and I think nobody's clear on what they're doing, both the students and the person who's speaking.” Carmen (service user educator) suggested that the “onus” of establishing, or communicating, clear expectations should be placed on health professional educators.

I think that being transparent about what it is they're expecting from you when you come in to tell the story is really important; and that onus can't necessarily be on the service user... it has to be on the person who's inviting them in to really think that through, and to have a framework for what they can expect. Who is in the room? And, why are we asking you now? What is the context of why you've been asked?

Sara (health professional educator) acknowledged her responsibility to service user educators, however, she also described feeling somewhat unprepared to take on this role herself, suggesting that perhaps this was a “resource issue”:

I would say that, if I'm going to ask people to share their story, I probably have a responsibility to [help them prepare], but, it's not really traditionally part of my role. I don't even know if I have the expertise to do that. I'm an educator, so I have expertise in education theory and pedagogy, and all that stuff, but I don't know that I'm the right person necessarily to help someone hone in *how* to tell their story in a way that's impactful and meaningful to them and to students.

Glen (service user educator), on the other hand, cautioned that the process of refining one’s story at the interface of health professional education (or in tandem with health professional educators) is not without the risk of sanitization, or the loss of a sense of authenticity:

One of the things that I find is by the time people have a really good grasp on their story, they’re academized [sic] and, is that a word? And so far from the street, and so far from the experience, that they've absorbed—they've been absorbed—into the textbook mindset.

#### Perceived versus actual work involved in storytelling

The work required to process experiences, compose a story and tell that story in a health professional setting was not always acknowledged, or perhaps recognized as such. In particular, some service user educators described perceived discrepancies between health professional educators’ understandings of—and the actual—work involved in storytelling. Fred described one such discrepancy whereby despite taking his role as a service user educator “very seriously” (i.e., “you have to take it seriously, because as a teacher or educator, it’s a serious thing to affect a student’s mark, or their profession, or their path in life”), he recalled being met with the well-intentioned remarks of a health professional educator who: “tried to make me understand that ‘you can't fail at this. You know there's no high expectations of you or anything like that, we just want you to share your story.’” In a similar instance, despite placing great importance on being prepared and organized, Sally’s (service user educator) concerns were seemingly dismissed with the reminder, “‘[Sally], you're telling *your* story, nobody knows it but you.’”.

#### The emotional work of storytelling

The sort of work most often described by both service user and health professional educators alike, was the uniquely emotional work of telling stories. For some, the emotional work of storytelling involved “managing anxiety,” while others attributed it to reliving difficult or “triggering” experiences. Sally (service user educator), recalled that arriving at the decision to share her story publicly was a lengthy and emotional process:

It took two or three years to be able to share my story... I did some sharing with [my peers]. You know, we had each other, kind of. Writing the story, sharing the story, and then supporting each other after we’d done the presentation.

Fred (service user educator) who described storytelling as “very draining”, stated that, “sharing your story… it can bring up bad things, and I think one time…it made me a little sad…it makes you look at, you know, ‘me’.” Elliot (service user educator) suggested that considerable thought be given to this aspect of storytelling: “It's difficult for people. You're asking people to do things that trigger [them]; it could actually be detrimental to their health leading up to and presenting.” Anthony (health professional educator) made a point of discussing the risk of “re-traumatization” with all of the service user educators he invited to speak to his students. In his words, when service user educators “go back down that path, [they] ‘open up that door’ because [they] feel like it's part of [their] recovery journey” but there’s “the potential for their re-traumatization.”

Some participants also spoke about managing the emotions of the student audience and other listeners. Glen (service user educator) described this as a balancing act between inspiring “deep level change” and maintaining “emotional containment.” He stated, “we have to deliver enough content in a memorable experience, but not blow their minds. So, [the course coordinator and I are] searching together for that balance.”

#### Emotional work as a downside of storytelling

It was generally accepted by participants in this study that storytelling necessitates emotional work. However, several participants alluded to this emotional work as being one of the drawbacks of SUI, and questioned whether this had to be so. In Elliot’s (service user educator) words:

Re-thinking things that you don't think about every day, because you don't want to... Your past breakdowns or your past psychoses or your past failures or successes, even that is emotionally taxing. And for people with a mental health disability, those strong emotions can actually make them worse. So that also, that's one of the drawbacks.

Sara (health professional educator) shared her suspicion that the emotional work of storytelling may actually impact service user educators’ decisions not to return when invited back; or to set boundaries for their involvement when doing so. She recalled the words of one service user educator who said:

I want to come back, but I will not talk about my father's suicide the way I did last year. It took me too long to... it brought up too much for me. So, I will come back and speak to your students, but I can't speak about that stuff.

Carmen described her efforts to reduce the emotional toll of storytelling in her role as a service user educator, explaining that over time she has made a conscious decision to tell less of her personal story, and rather, focus on concepts, theories, and values that have been identified as important by service user educator communities. She described her approach by saying:

I go in and talk to the [health professional] students; I tell them a little bit of my story. As time has gone by, I say less and less actually... I really, you know—I give them the broad strokes.

Several participants identified support or ‘follow-up’ after storytelling as another important strategy for mitigating the negative impacts of the emotional work of storytelling, though as it was described, this remains a largely unmet need. Sara (health professional educator) shared:

As an educator I think about what it does to the students, and I'm always thinking that... but I don't typically worry about the mental health of my speakers, and... sometimes people will get emotional... it will bring up feelings from their past... none of that is a bad thing, but we don't really... we don't really do any follow-up. We kind of say, “thank you so much! That was lovely!”

### Broadening conceptualizations of service user educators’ knowledge: “I have knowledge that can help them in their actual practice”

#### Stories as the unique knowledge of service users

Participants accounts revealed tensions related to whether individuals’ lived experiences or personal stories and service user knowledge can be considered one in the same, and raised important questions about the different kinds of stories to be told. Stories were described as the “unique knowledge” (Sara, health professional educator) of service user educators, and in particular, as the sort of knowledge that health professional educators are unable to offer. Rita (health professional educator) asserted that “[service user educators’] firsthand accounts are really an important part of what the students need to hear.” She explained that, “consumers are really effective in helping [students]… in ways that [she], or other faculty, could not.” Lindsay (health professional educator) echoed this, saying, “[service user educators] offer something that we can't.”

#### Service user knowledge as more than ‘life stories’

Despite many participants’ motivations to share their stories to elicit change (as described above), it was not uncommon for participants to recount experiences of storytelling that involved, “a whole session” wherein one service user educator, “just tells his whole story. You know, how his mental illness influences his ability to function in daily life, and his work, and all that kind of stuff” (Lindsay, health professional educator). Heather (service user educator) noted that she and fellow service user educators, “just went in and told [their] story…and answered a bunch of questions afterward.” Likewise, Kimberly (service user educator) said of her contribution:

So, it’s like a life story, it’s not really a lecture. It’s more stories... I talk about my childhood, I talk about high school, I talk about university, I talk about meeting my husband, my family’s involvement in my care, and then where I am at now with work.

Several participants offered critiques of such practices, calling for a broader conceptualizing of service user educators’ knowledge, beyond stories of (overcoming) mental distress, diagnosis or treatment. Joel (service user educator) stated:

There's this idea that what we're sharing with [students] is our perspective, and giving them a window into our experience; and yes, that's great, that's good... But actually, you know what? I think I have knowledge that can help them in their actual practice... There's really knowledge out there; it's not just about listening to someone tell their story of being admitted [to hospital], or being restrained...”

In response to such criticisms, Carmen, a service user educator with experience in coordinating SUI, described trying to recruit service user educators who were able to offer insights beyond their stories of ‘illness.’ She recalled:

We had people who were able to couch their narrative a bit... so, we didn't get the ‘play- by-play,’ but you were able to get a bit of an understanding of why they said what they said. We were looking for people who had an analysis that was broader than their own situation. So, people who talked about, for example, coercion or justice; people who talked about the social determinants of health; people who talked about power and privilege in some way, and either pertaining to their own, or what they saw in people who had helped them or hurt them.

#### Service user knowledge beyond storytelling

Fred (service user educator) offered one example of sharing his lived experience outside of the realm of personal storytelling to educate students, wherein he drew from his firsthand knowledge of the lesser-known community-based mental health resources (e.g., “a local church”), to act as a “facilitator” in systems navigation. Fred explained that his goal was to, “try and get the students to think outside the box a little bit; to utilize more services that are available. Because, often the [government-run] services are limited.”

Some health professional educators also suggested that service user educators’ knowledge contributions should extend beyond telling “life stories,” to include, for example, a more focused discussion of a particular concept or practice approach, or a focus on service user educators’ unique areas of expertise. Rita (health professional educator) described this as, “a much more focused approach…than just telling; no, I won't say *just*, but telling a story of one's life. It's really looking at a particular approach in [therapy] and talking about how that facilitated [their] growth.” Sara (health professional educator) spoke about treating service user educators’ knowledge as a sort of “lived experience expertise,” versus a life story; asserting:

We can't just have people come in and share their experiences. I think we need to treat [service user educators] like we treat any other speaker, and you know we're asking people who are experts at whatever we're asking them to speak about, and whether they're clinicians or- whether it's clinical expertise, or lived experience expertise. So, treating them not as just a story, but actually saying- inviting them to be part of the curriculum, in a meaningful way.

## Discussion

While storytelling was described by participants as central to the way service user educators convey their knowledge in the context of health professional education, our findings illuminate some of the complexities in using storytelling as a means for sharing service user knowledge. The discussion is framed around three important tensions made visible through the study: (i) the performance and consumption of stories (Voronka, 2019); (ii) the emotional labour of composing and telling stories (Brosnan, 2019; Hochschild, 1979; Oksala, 2016; Voronka, 2019); (iii) and epistemic injustice (Fricker, 2007; LeBlanc & Kinsella, 2016) as it relates to the *kinds* of stories that seem to be welcomed into health professional education spaces. The discussion of these issues is followed by a set of reflexive prompts for educators and other stakeholders interested in critical engagement with service user educators in health professional education.

### Performance and the consumption of service user educators’ stories

Both service user and health professional educators described storytelling in the context of SUI as ‘powerful.’ In particular, participants linked storytelling to popular forms of performance and credited the performative or evocative nature of service user educator storytelling with deepened student engagement and memorable learning experiences. The ways in which this element of storytelling was regarded, however, seemed to differ between and within these stakeholder groups. Some participants problematized the expectation that service user educators should ‘perform,’ or share particularly moving stories as a means for conveying their experiential knowledge; with one participant describing this work as “emotional pornography,” (Glen) and another describing the practice of soliciting emotional stories to enhance student learning as “perverse” (Sara). These findings resonate with what Costa et al. (2012) describe as ‘patient porn,’ in their discussion of the interactive nature of service user storytelling, explaining that, “while some people reveal their most intimate personal details, others achieve relief through passive watching” (p.86).

Our findings also lend support to discussions of the performance and consumption of service user educators’ stories as ‘commodities,’ and in particular, the “commodification of Otherness” (Hooks, 1992 as cited in Voronka, 2015, p. 261). As Razack (1993) explains, expecting service user educators’ stories to be powerful or moving (read: entertaining), risks shifting the focus to one of student engagement, rather than compelling students and other listeners, to, “explore their own complicity in the oppression of others” (p. 66). Voronka (2017) has urged Mad scholars, activists and service user educators to continue to reassess the ways in which marginalized identities are mobilized and enacted, “when commodifying our experiences within the systems that sustain our subjugation” (p. 337). For instance, when service user knowledge is solicited for the purpose of illuminating a concept, theory, or practice with roots or origins in professional knowledge (i.e., underpinned by biomedical models for understanding mental distress), storytelling may actually serve to reinforce dominant narratives (Voronka, 2016).

Several participants described being invited to speak to students about their experience of a particular psychiatric diagnosis and their subsequent ‘recovery’ (typically facilitated by health professional intervention) for the purpose of enlightening students about the *why* behind service users’ more ‘difficult’ behaviours (e.g., non-compliance or frequent ‘no-shows’). In this way, service user educators’ stories of Otherness are often consumed by health professional education students as a “‘teaching tool’ and ‘learning material’, where they become objectified and commodified as ‘a living textbook, a *means* (of learning) *to an end* (of greater competence)’” (de Bie, 2021, p. 9). Rather than spark transformative, systems-level change, such stories risk supporting, confirming, or reinforcing dominant discourses, which maintain the status quo. Although these may make for entertaining, or ‘good stories,’ there is risk that their telling may simply inspire more storytelling, rather than political or social change (Polletta, 1998).

Within health professional education, service user educators have not always been regarded as active epistemic agents or *knowers* (see section on *Epistemic Injustice* below for elaboration), but rather were studied as objects to *know about* (Costa et al., 2012). Without control over the context in which a story is told, or over the gaze of the audience (i.e., typically a psychiatric or medicalized gaze), service user educators may find themselves faced with an audience who is unable to accurately interpret their intended message (Voronka, 2019). Health professional education students, for instance, may not possess the alternative epistemic resources (i.e., language, concepts, and theories developed by/within service user and Mad communities) required to understand some critical stories as they are intended. So, despite service user educators’ best efforts to share their individual and collective knowledges, their stories may come to be understood within the discursive confines of dominant ‘mental illness’ discourse, with their intended meanings altered or skewed (Voronka, 2019; Voronka & Grant, 2021).

It is vital that careful consideration be given to creating conditions whereby service user educators have power over the knowledge being produced and shared; not simply over the content of their stories, but also the broader contexts in which their stories will come to be understood and interpreted (O’Donnell et al., 2019). As de Bie (2021) has recently suggested, SUI oriented toward social justice requires consideration of, “a more expansive, service user- informed ethics for engaging with first-person accounts,” where our focus is less on whether students enjoy, learn, and gain a greater sense of empathy from their engagement with these stories, and more on how we might ethically engage with these stories and the service user educators and communities we teach alongside.

### The emotional labour of storytelling

Participants’ accounts of the work required to craft and share stories is consistent with recent literature discussing the emotional or affective labour of SUI (Brosnan, 2019; Voronka, 2017). Brosnan (2019) suggests that acknowledging the emotional labour of service user educators—which they contend, “is often silenced, unacknowledged, and invisible”—is ethically and politically imperative (p. 2). Brosnan argues that despite notable contributions (Church, 1995; Church & Reville, 1988; Voronka, 2017), little attention has been paid to the emotional or affective costs of involving service user educators in health professional spaces (Brosnan, 2019). *Emotional labour* is described as an immaterial form of labour which involves, “…the management of feeling to create a publicly observable facial and bodily display,” (Hochschild, 1983, p. 7), and shares similarities with *affective labour,* wherein, “workers are expected to mobilize emotional and social skills for professional goals, resulting in the blending of the private and the public” aimed at producing *affects* (Oksala, 2016, p.284). Several participants in this study described managing emotions and affects in themselves and others as part of the work of service user storytelling.

Perhaps the clearest example of emotional or affective labour in this study can be found in participants’ accounts of a perceived expectation and/or personal desire to perform or “model wellness” in order to quell fears or instill in their audience a deeper sense of empathy, compassion, and humanity. Several participants specifically linked this performance of wellness to the goal of ‘humanizing’ themselves and others who have experienced mental distress. These findings exemplify Voronka’s (2019) contention that service user storytelling as a means to address the problems of stigma and discrimination places responsibility on service users to diminish the discrimination they experience. Voronka (2019) explains that, “by sharing our stories with others who may discriminate against us. In effect, to counter dehumanization, it becomes our job to share our stories in attempt to humanize ourselves” (p. 13). In other words, service user educators perform a sort of emotional or affective labour to produce (or elicit) feelings of sympathy, compassion, and understanding in students and faculty, so that they might view service users as “redeemable subjects worthy of pity and investment” (Voronka, 2015, p. 300).

Brosnan (2019) describes a certain emotional labour required in communicating stories in contested—sometimes hostile—health professional spaces, where service users are not necessarily regarded as equal knowers, or even as bearers of valid knowledge. It is possible that the presumed emotionality of this work precludes service users’ knowledge from being viewed as valid (Brosnan, 2019). Furthermore, service user educators may be required to navigate difficult emotions and possible ‘re-traumatization’ for the purpose of upholding appearances of rationality, composure, and stability in order to convey oneself as a legitimate knower (Brosnan, 2019). Despite the emotional dimensions and products of this work—described by some participants as what makes storytelling particularly powerful—many also identified the emotional or affective labour of storytelling as one of the downsides of this work. Several participants described strategies for managing the emotional toll of storytelling, such as establishing supportive networks and opportunities for debriefing, or telling less in the way of intimate personal details, and setting firm boundaries related to the content of their stories. For some participants, a shift away from overtly emotional storytelling meant speaking more to societal issues and injustices informed by and/or grounded in experiential knowledge.

There appeared to be some discrepancy between the actual labour undertaken by service user educators in sharing their stories and the perceptions of health professional educators in recognizing the magnitude of this work. The emotional labour, and other work involved in storytelling by service user educators, appeared to be largely invisible to, or at least minimized (though perhaps unknowingly, on account of its invisibility) by those soliciting stories (Brosnan, 2019). The invisibility of this labour may offer some insight into why this work is seldom fairly remunerated. This invisibility was also reflected in the casual manner participants spoke about service user educators “having a story” to tell; as if to suggest that by virtue of having direct/lived experience(s) with the mental health system that one automatically possesses a singular, coherent, or even intelligible story to be readily shared with health professional students. This contrasted with some participants’ reports of a complex and laborious process involved in both storying and telling their knowledge. Participants described attempts to condense years of lived experience into a coherent and impactful story; making difficult decisions about which anecdotes would have the greatest impact on students (i.e., deciding what is most likely to “inspire deep-level change”); navigating risks of re-traumatization; and managing anxieties related to storytelling to a student audience, and/or the ways their story would impact students’ professional approach.

### Epistemic injustice: which stories are (not) being told?

Most service user educators described invitations to share personal (or ‘life’) stories detailing their lived experiences of mental illness and recovery within health professional programs. These were stories that might offer students “a window into their experience,” and insight into *why* service users might engage in particular behaviours within clinical interactions (e.g., non-compliance or missed appointments). Such invitations seemed to contrast with many service user educators’ described motivations to tell their stories as a means to address systemic issues (e.g., justice, coercion, or discrimination); impart practical wisdom regarding systems navigation; and effect change in the mental healthcare system (e.g., reconceptualizing trauma, or instilling a greater sense of empathy and compassion in future health professionals).

This apparent disconnect between the kinds of stories service user educators wish to tell, and those they are invited to tell, resonates with recent literature highlighting the epistemological implications of soliciting service user educators’ stories for use in health professional education; particularly, the perpetuation of epistemic forms of injustice (de Bie, 2021). Service user educators’ knowledge represents a form of marginalized (or marginally situated) knowledge in health professional education contexts given the dominance of ‘professional’ knowledge. As such, the concept of epistemic injustice provides a generative theoretical perspective for thinking about the ways in which service user knowledge has come to be—and in some cases, remains—suppressed or marginalized within these spaces (Dotson, 2011; Fricker, 2007; LeBlanc & Kinsella, 2016; Pohlhaus, 2017). Epistemic injustice refers to the distinct wrong done to someone in their capacity as a *knower*; restricting their ability to engage in the basic everyday practices of knowing, conveying knowledge to others, and/or actively participating in the production of a collective knowledge base (Dotson, 2011; Fricker, 2007; Pohlhaus, 2017). Engagement with this concept gives rise to questions such as, what constitutes valid knowledge? Who are deemed ‘legitimate knowers’? And whose knowledge should count?

Several participants called for broader understandings of service users’ knowledge than that of ‘life stories’ centered around overcoming mental illness, or *mental illness narratives* (de Bie, 2021). In this way, our findings complicate the uncritical inclusion of service user educators’ stories by drawing attention to the knowledge that may be overlooked, suppressed or excluded from health professional education spaces in the *kind* of stories being invited and told. Costa et al. (2012) observed that the use of service user educators’ stories have moved away from the origins of storytelling by psychiatric survivors, in that rather than contributing to radical change, they are being used to “further solidify hegemonic accounts of mental illness” (p.87). While such stories may inform health care practices in important ways, Costa et al. (2012) contend that:

“if we listen only for the ‘lived experience’ of individuals, and only for processes of illness and recovery—we will miss many other vital storylines. We need to complicate what we are listening for: to listen less for stories of healing and recovery and more for stories of resistance and opposition, collective action and social change” (p.96).

When service user educators’ mental illness narratives are uncritically solicited, alternative (and typically more radically marginalized) knowledge (e.g., stories of resistance or survivor activism and collective action) is at risk of being excluded or overlooked, constituting a particular form of epistemic injustice known as *contributory injustice* (Dotson, 2012; Pohlhaus, 2017). Contributory injustice occurs through the systemic exclusion or dismissal of the knowledge and language developed within marginally situated communities by those situated more dominantly (Miller Tate, 2019, p. 97). When applied to the findings of this study, we see that the potential for the perpetuation of contributory injustice is both complex and insidious. This is because not *all* service user educators’ stories are denied uptake within health professional education.

The sharing of life stories and mental illness narratives through SUI suggests that service user educators are indeed *contributing* knowledge to health professional education. However, the way in which service user educators are typically invited to participate (e.g., ad hoc guest lectures), and the *kind* of stories that are solicited (e.g., mental illness narratives), may actually be in tensions with the political aims of broader service user, psychiatric survivor and Mad communities, as stories more closely aligned with these aims are effectively overlooked and/or excluded. As de Bie (2021) has pointed out, some stories are privileged over others, “arbitrating the value of stories based on student enjoyment, prioritising the learning needs of non-Mad students and failing to recognise the contribution of personal narratives to collective Mad/survivor expertise” (p. 9).

Life stories and mental illness narratives are typically told using dominant epistemic resources (i.e., language, concepts, theories) espoused by health professional educators and their students, whereas stories of resistance more often rely on marginalized epistemic resources, (e.g., Mad epistemologies or critical understandings of concepts like recovery), and are less likely to be readily received, or even understood, by health professional audiences. So, while marginally situated knowers are often able to make sense of and articulate aspects of their experience relatively effortlessly among themselves, they remain unable to communicate this knowledge with the same ease or effectiveness in mainstream discourse (Dotson, 2012). As a result, some forms of service user knowledge (e.g., resistance narratives) may remain suppressed, despite appearances of service user inclusion. Voronka (2015) cautions that when storytelling is approached as an “inclusionary practice” (p. 273), whereby exclusion is positioned as the problem in need of redress, larger structural issues of inequity and injustice are at risk of being left unchallenged.

When understood through a lens of epistemic injustice, our findings reflect issues regarding the legitimacy of service user educators' stories as a source of knowledge, and whether or not these stories are viewed as contributing to a collective knowledge base (de Bie, 2021). Costa et al. (2012) have troubled the assumption that individual stories can, “single-handedly change deeply embedded, oppressive and interconnected powerful social structures” (p.98). They have called for a reclaiming of stories as political knowledge and encouraged those “who reveal their stories to consider doing so in a way that is politically accountable and focused on social justice change” (p. 99). Such social accountability may result in storytelling that is more closely aligned with the vast body of work by psychiatric survivors, service users, Mad activists, scholars, and their allies. Informed by individual and collective experiences, this diverse group has worked to advance alternative epistemological bases and approaches to responding to mental distress and diversity (Beresford & Russo, 2016; LeFrancois et al., 2016; Newbigging & Ridley, 2018).

### Reflexive prompts for educators

While prioritizing service user educators’ involvement in health professional education through storytelling will not inevitably lead to epistemic and social justice outcomes, attending to the ethical and epistemic complexities of this practice through thoughtful engagement in critical reflexivity stands to support us in this pursuit (de Bie, 2021). Critical reflexivity, not to be confused with personal reflection, involves careful interrogation of the grounds upon which taken-for-granted, or normative, claims about knowledge are generated and accepted, along with the situated perspectives from which knowledge claims are produced (Harding, 1991; Kinsella & Whiteford, 2009). We propose the following series of critically reflexive prompts (Table 1) for educators looking to attend to the complexities inherent in this work toward greater ethical, social and political accountability to service user educators:

**Table 1:** Reflexive Prompts for Educators

Aim	Reflexive prompt
Troubling expectations that service user educators should entertain	Is it possible that my motivation for soliciting a service user educator’s story is to make for a more memorable or engaging lesson?
	Is it my expectation that service user educators’ contributions will be engaging or entertaining?
	How might my expectations around service user educators’ involvement (regarding performativity) differ from those I have for non-service user educators?
Acknowledging the extent of service user educators’ emotional and/or affective labour	How important is it to me that service user educators share intimate personal details, diagnoses, or firsthand accounts of their experiences with mental health services?
	Is it possible that I am asking service user educators to do the emotional/affective labour of ‘humanizing’ or presenting themselves as “redeemable subjects” (Voronka, 2015, p. 300)?
	Has my determination of the remuneration or compensation I am prepared to offer service user educators taken into account the extent of the emotional and epistemic labour involved in their contribution?
Mitigating epistemic injustice	Have I discussed the legitimacy of service user-produced knowledge with students?
	Have I engaged with the concepts and ideas presented in service user/survivor-produced literature, toward establishing a conceptual foundation which would enable students to interpret service user educators’ storied knowledge as it is intended?
	Have I discussed systems of oppression and privilege (e.g., sanism/sane privilege) with students?
Fostering supportive (epistemic) environments	Is my decision to involve service user educators in my [classroom, program, activity] supported by my department? If not, why might that be?
	What steps might can I take to create safer, more supportive epistemic conditions for service user involvement and storytelling?
Supporting epistemic communities	How can I encourage ‘affinity groups’ among the service user educators in my network?
	Have I established connections with local service user/survivor collectives or communities to inquire about individuals or groups who may be interested in the role of service user educator?
Critical/Mad (positive) pedagogy and transformative learning	Have I thought about the involvement of service user educators in health professional education classrooms as a critical pedagogical method?
	Is my aim in involving service user educators in [health professional education context] to trouble taken-for-granted health professional knowledge and practices toward transformative learning? Or, to provide students with an exemplar of popular health professional (e.g., biopsychosocial) concepts or theories?
	Am I able to clearly communicate these objectives to service user educators? Am I open to service user educators’ feedback regarding these objectives?

## Limitations

The study was situated in a bounded context, focused primarily on service user and health professional educators from one health profession. While the findings are therefore not generalizable, the insights may hold resonance and be practically transferable to other health professional education contexts. It is also possible that the participants involved in this study were over-representative of those who have had positive experiences with SUI, as the majority reported multi-year tenures with health professional education programs. It is presumed that the participants in this study may have experiences that differ from those who discontinued involvement soon after initial involvement. As such, it is important to acknowledge that the findings of this study may not be representative of the diversity of experiences of service user educators.

## Directions for further research

Consistent with suggestions by de Bie (2021), this research points to a need for further research exploring ways we might bridge the gap between service user educator, Mad, consumer/survivor/ex-patient (c/s/x), and health professional education communities. Research which considers dominant ideologies and contemporary approaches in health professional education, and/or seeks opportunities to increase awareness of Mad studies and critical disability studies would be fruitful. This research points to a need for the development of SUI approaches aimed at fostering ethical and epistemically just conditions for service users. For example, approaches which prioritize setting the stage for SUI through the use of ‘priming concepts’ drawn from Mad studies and other service user-produced literature. Bryant (2020) has suggested that “bringing people together to create shared stories avoids some of [the] risk” of reproducing damaging stereotypes and being misunderstood (p. 317). Future research might also focus on supporting the development and sustainability of service user educator communities of practice toward establishing a collective, more socially accountable service user knowledge base.

## Conclusion

In this paper, we point to a need for greater critical reflexivity related to *how* and *why* storytelling by service user educators is conceptualized and enacted in health professional education. This work contributes to emerging conversations around the complexities inherent in this work, and supports recent findings in a growing body of literature which suggests that while the inclusion of service user educators’ stories can be both important and meaningful, their use in health professional education is not without risk. Our findings trouble the notion that storytelling in the context of SUI is a wholly positive or benevolent endeavour, and offers a set of critically reflexive prompts in hopes of engaging the imaginations of educators interested in more ethical and epistemically just approaches to this practice.
